# Change of force and lever arm of the hip abductors after subtrochanteric de‐/rotational osteotomy

**DOI:** 10.1002/jeo2.70274

**Published:** 2025-06-05

**Authors:** Christoph Zindel, Patrick O. Zingg, Michel Meisterhans, Samuel Haupt, Armando Hoch, Andreas Flury

**Affiliations:** ^1^ Department of Orthopedics, Balgrist University Hospital University of Zurich Zurich Switzerland

**Keywords:** 3D analysis, abductor force, subtrochanteric osteotomy

## Abstract

**Purpose:**

Previous studies have shown that rotational osteotomy of the femur reliably corrects anatomical torsion but may result in a ±1 cm antero‐posterior shift of the greater trochanter (GT) per 10° of torsional correction. It is unclear whether an inadvertent displacement of the GT following surgical treatment of femoral maltorsion affects the hip abductors in a clinically relevant manner and needs to be addressed by compensatory digastric trochanteric osteotomy. The aim of this study was to investigate the influence of rotational subtrochanteric osteotomy on hip abductor force generation using three‐dimensional (3D) surface models.

**Methods:**

A 3D statistical shape model (SSM) of a hip/femur was used. From a baseline value of 12°, the SSM was derotated/rotated in 10° increments, creating five different scenarios of anatomical femoral torsion: 32°, 22°, 12° (baseline), 2°, −8° (corresponding to 8° of retro torsion). The origins and insertions of the gluteus medius (GMed) and minimus (GMin) muscles were created. The hip abductor moment was defined as the muscle force (of the hip abductor muscles) multiplied by the force ratio in the direction of abduction (FRDA) multiplied by the lever arm. All values were measured, whereas muscle strength was defined as the length of the muscle fibres in relation to their optimal length.

**Results:**

Results indicated minimal changes in muscle length (±1.2%) and FRDA (1.7% to −7%) for GMed und GMin across all scenarios. However, lever arm demonstrated variations (21% to −10%), with an increase observed for derotational osteotomies and a decrease for rotational osteotomies.

**Conclusion:**

Subtrochanteric osteotomy in the management of femoral maltorsion affects the hip abduction moment solely through the altered lever arm. A 20° derotation for increased femoral torsion (FT) corresponds to a 20% increase in abduction force of the GMed (improved lever arm), whereas a 20° rotation for decreased FT reduces the abduction force by 10%.

**Level of Evidence:**

Level III, diagnostic.

**Abbreviations**
3D: three‐dimensional
FRDA: force ratio in the direction of abduction
FT: femoral torsion
GMed: gluteus medius muscle
GMin: gluteus minimus muscle
GT: greater trochanter
SSM: statistical shape bone model

## INTRODUCTION

Torsional abnormalities of the femur are thought to be a potential cause of hip pain [27]. Patients with decreased femoral antetorsion or retrotorsion present with decreased internal rotation and anterior femoroacetabular impingement [3, 16]. Conversely, excessive femoral antetorsion in the hip may lead to overloading of the anterosuperior acetabular labrum and cartilage due to partial exposure of the femoral head [15, 27], and has been recognized as an additional cause of ischiofemoral hip impingement [25]. Geometrically, it is associated with a reduced abductor lever arm with increased joint reaction force and overuse of the hip abductors leading to lateral hip pain [24].

Anatomical femoral torsion (FT) is based on the centre of the femoral neck as the reference point, whereas FT is based on the greater trochanter (GT). According to the literature, the physiological value of FT in adults is approximately 12° ± 10° [26]. Promising results have been reported with surgical correction of maltorsion to anatomical values in symptomatic patients [13, 23, 25]. However, concerns have been raised about malcorrection of the GT following subtrochanteric osteotomy, due to the geographic relationship of the GT and the degree of FT [2]. This natural compensatory mechanism works in such a way that the functional FT is reduced by the anteroposterior position of the GT to the value of femora with normal anatomical FT [7], thus improving the course of action and partially compensating for the lever arm. Therefore, by rotating the centre of the femoral neck out of the mechanical conflict zone while aiming for anatomical FT values, the GT, as the insertion point of the hip abductors, does not experience a normalization of its position, but rather an overcorrection [2, 7]. This potentially affects the hip abductor force vector, reducing the lever arm and the hip abductor moment, leading to muscle fatigue and lateral hip pain.

To date it remains unclear whether an average 1.5 cm displacement of the GT due to a 15° torsional correction affects the hip abductors in a way that is clinically relevant [7] and needs to be addressed by a compensatory digastric trochanteric osteotomy.

The purpose of this study was to investigate the influence of rotational/derotational subtrochanteric osteotomy on hip abductor force generation using three‐dimensional (3D) surface models. We hypothesized that (1) derotational osteotomy for excessive FT will increase the lever arm of the hip abductors, whereas increasing FT will decrease it, but (2) the change in hip abductor muscle length and force ratio is small, making the effect of maltorsion‐correcting osteotomies on hip abductor force generation negligible.

## MATERIALS AND METHODS

A previously published 3D statistical shape bone model (SSM) of 61 femora and hips from routine postmortem CT scans was used [6, 19]. The CT data allow for image segmentation to create 3D triangular surface models, as outlined in previous studies [9, 12, 29, 30, 31]. The SSM was then developed using a non‐rigid registration algorithm and 20 principal components were defined to represent 99% of the shape variance [18]. The 3D models were postprocessed in the in‐house 3D planning software (CASPA; Balgrist CARD). First, the 3D femoral surface model was aligned with the reference points according to the guidelines of the International Society of Biomechanics (ISB) [28], resulting in a true coordinate system for the hip including the coronal plane (ap) and the axial plane. The centre of the hip joint was defined as the centre of a sphere fitted to the surface of the femoral head.

### Origin and insertion of the hip abductors

**Figure 1:**
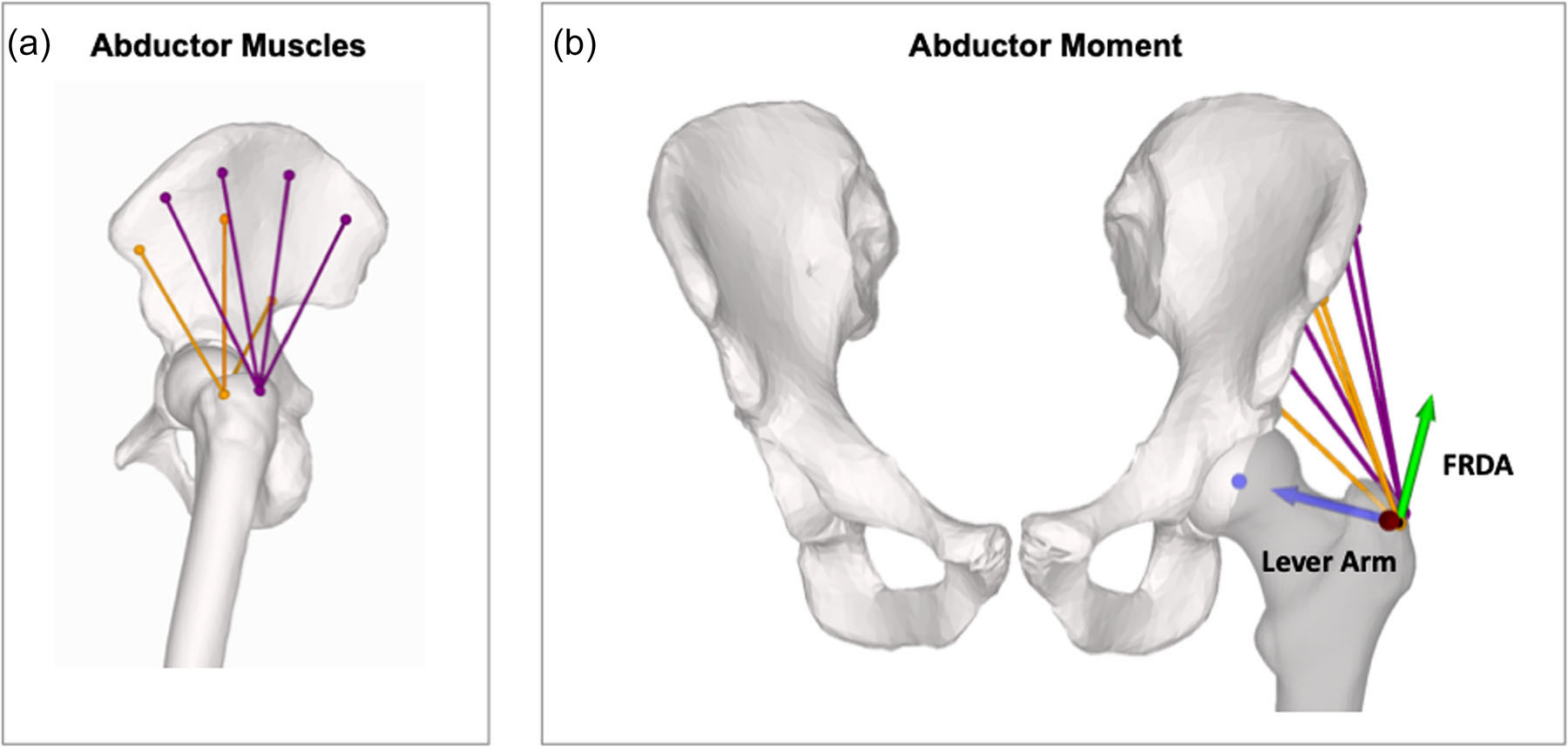
(a) Hip abductors divided into separate subbranches (gluteus medius in purple and gluteus minimus in orange). (b) Force vectors of the hip abductors. The hip abductor moment is calculated as multiplication of the muscle force (of the hip abductor muscles) multiplied by the force ratio in the direction of abduction (FRDA—green arrow) multiplied by the lever arm (blue arrow). FRDA, force ratio in the direction of abduction.

The origins and insertions of the gluteus medius (GMed) and minimus (GMin) muscles were defined according to the literature [5]. The accumulated direction of the muscle force was simulated as a string between the origin and insertion. Due to the fan shape, GMed was divided into 4 (anterior, anteromedial, posteromedial and posterior) and the GMin into 3 (anterior, medial and posterior) separate origins, leading to four and three subbranches of these muscles, respectively (Figure 1a).

### Anatomical and functional torsion

**Figure 2:**
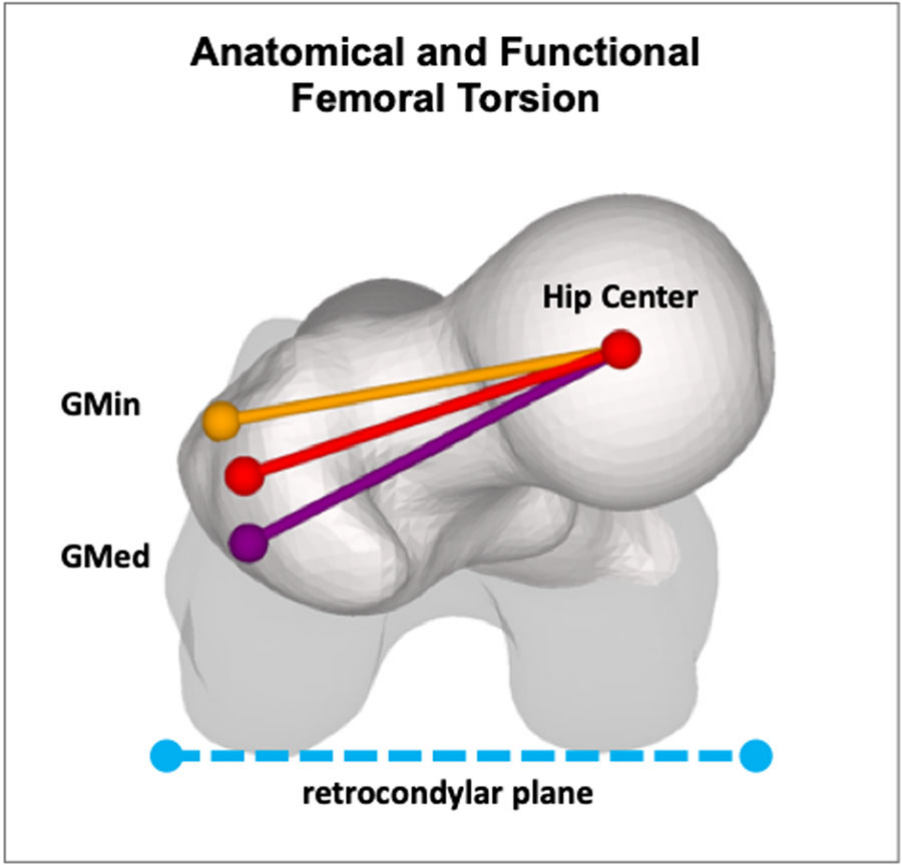
Anatomical femoral torsion is defined as the two‐dimensional angle between the femoral neck axis (shown in red) and the retrocondylar plane (shown in blue), projected onto the axial plane of the hip coordinate system. In contrast, functional femoral torsion uses the greater trochanter centre instead of the femoral neck centre as an anatomical reference (not shown). The lever arm (averaged across all subbranches) of the gluteus medius muscle (GMed) is illustrated in purple and that of the gluteus minimus muscle (GMin) in orange.

The 3D anatomical torsion measurement followed a method similar to Murphy's technique [20]. The centre of the hip and the centre of the femoral neck were determined. Anatomical FT was determined as the two‐dimensional angle between the femoral neck axis (a line connecting the centre of the femoral head to the centre of the femoral neck) and the retrocondylar plane, projected onto the axial plane of the hip coordinate system. Functional FT is determined as a two‐dimensional angle projected onto the axial plane, similar to anatomical torsion, but with the centre of the GT instead of the femoral neck centre as an anatomical reference. In this study, functional torsion per se was not investigated. Instead, the relevant muscles inserting at the GT (GMed and GMin) were analysed given their biomechanical relevance (Figure 2).

### Simulation of subtrochanteric osteotomy

**Figure 3:**
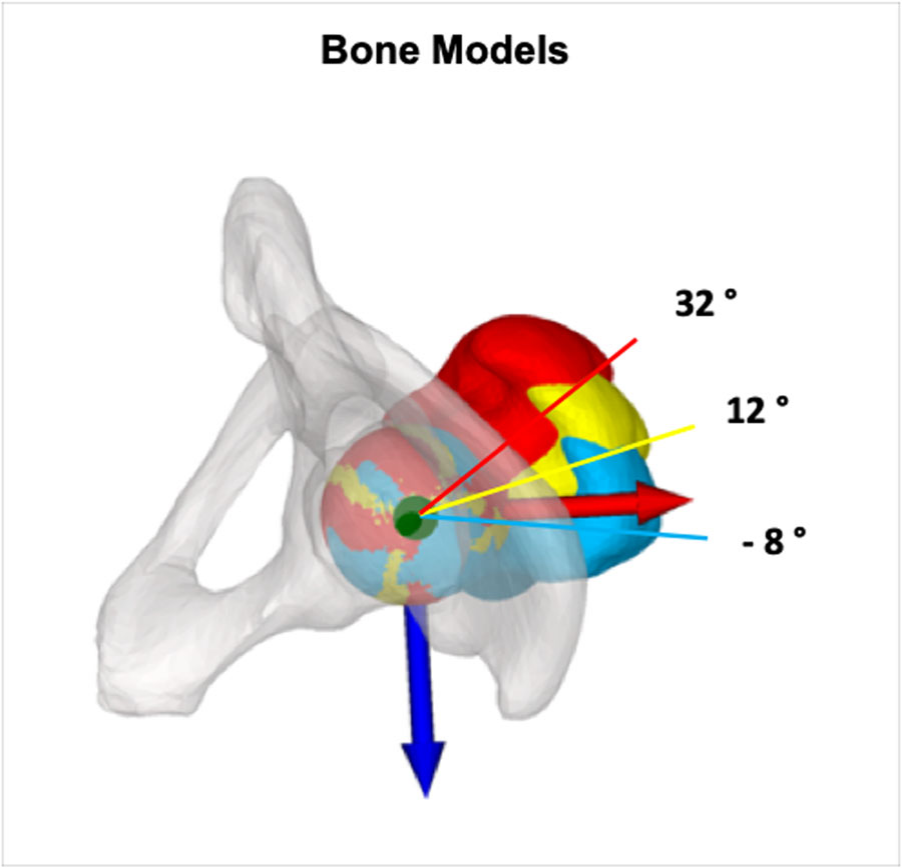
Axial view showing simulated anatomical femoral torsion of 32° (red), 12° (yellow) and −8° (blue).

From a baseline value of 12°, the SSM was derotated/rotated in 10° increments, creating five different scenarios of anatomical FT: 32°, 22°, 12° (baseline), 2°, –8° (corresponding to 8° of retro torsion) [14] (Figure 3). A subtrochanteric osteotomy was then simulated to derotate (FT decrease) or rotate (FT increase) of the malrotated femurs back to normal anatomy (12° antetorsion). To simulate the subtrochanteric osteotomy, a 6‐hole 4.5 LCP plate was fixed to the lateral cortex of the femur, with the centre of the plate determining the location of the osteotomy. The osteotomy was made perpendicular to the mechanical axis [8]. To maintain consistent anterior knee alignment (with the patella facing forward), the proximal part of the femur was rotated rather than the distal part. By shifting the distal part to the proximal part of the femur, the lateral cortex was realigned to ensure optimal positioning of the LCP plate. Finally, the femoral head was repositioned (shifted) back to its original position to ensure a constant centre of rotation of the hip.

### The hip abductor moment: Measurement of muscle length, lever arm and FRDA

The moment generated by a muscle force quantifies the potential of the muscle to induce rotational motion about a specific spatial line, known as the axis of rotation or screw axis. The moment arm refers to the distance associated with this moment of muscle force around the screw axis. In the model presented here, the axis of rotation passes through the centre of the hip and is aligned in the AP plane of the hip. The hip abductor moment is defined as the muscle force (of the hip abductor muscles) multiplied by the force ratio in the direction of abduction (FRDA) multiplied by the lever arm.

(1) $$M_{\mathrm{ABD}}=F_{\mathrm{ABD}}\times \mathrm{FRDA}\times L_{\mathrm{ABD}}.$$

Equation (1) calculation of the hip abductor moment. The hip abductor moment (*M*_ABD_) is calculated as the product of the total abductor muscle force (*F*_ABD_), the FDRA and the corresponding lever arm (*L*_ABD_).

Muscle strength depends on the length of the muscle fibres in relation to their optimum length [1]. Therefore, muscle force is directly related to the length of the muscle, which leads us to analyse only the length of the muscle. All muscles (and their subbranches) were analysed individually for each different FT scenario. Muscle length (in Equation 1 represented as *F*_ABD_) was measured as the distance from the origin to its insertion and reported in millimetres (mm). The change in muscle length was calculated in relative (%) and absolute (mm) numbers compared to the baseline model, thus accounting for muscle force. To calculate FRDA, a local coordinate system was centred at the point of insertion of the muscle of interest. It was oriented so that the *x*‐axis pointed to the hip centre and the *z*‐axis to the AP plane. The resulting *y*‐axis represented the directional vector of the FRDA (pointing in the direction of abduction motion), as shown in Figure 1b. The FRDA was calculated as a proportion of the total muscle force and expressed as a percentage (%). The change of the FRDA between the deformity (preoperative) to the baseline (postoperative) was reported in %. The lever arm (in Equation 1 represented as *L*_ABD_) was calculated as the distance between the muscle insertion at the GT and the centre of the hip (representing the centre of rotation) and reported in millimetres (mm). The change in lever arm was calculated as the difference in lever arm between two bone models being compared and was reported in absolute (mm) and relative (%) terms.

To represent the total muscle (of GMed and GMin, respectively), muscle length, FRDA and lever arm were averaged across all subbranches.

## RESULTS

Detailed results are shown in Table 1.

**Table 1:** Summary of results.

Muscle length, FDRA, lever arm								
	20° rotation		10° rotation		10° derotation		20° derotation	
	(mm)	(%)	(mm)	(%)	(mm)	(%)	(mm)	(%)
*Muscle length*								
**M. gluteus medius**								
Anterior	6.43	5.64	3.41	2.91	–3.66	–2.95	–7.46	–5.83
Anteromedial	0.72	0.57	0.59	0.47	–1.04	–0.81	–2.47	–1.91
Posteromedial	–5.94	–3.95	–2.97	–2.01	2.88	2.03	5.57	4.01
Posterior	–10.98	–7.21	–5.60	–3.82	5.68	4.19	11.27	8.67
Avg	–2.44	–1.24	–1.14	–0.61	0.97	0.62	1.73	1.24
**M. gluteus minimus**								
Anterior	8.03	8.52	4.17	4.25	–4.33	–4.07	–8.69	–7.83
Medial	–1.31	–1.16	–0.52	–0.46	0.23	0.21	0.16	0.14
Posterior	–6.66	–6.40	–3.48	–3.45	3.69	3.94	7.50	8.35
Avg	0.02	0.32	0.06	0.11	–0.14	0.03	–0.34	0.22
*FRDA*	(%)	(%)	(%)	(%)	(%)	(%)	(%)	(%)
**M. gluteus medius**								
Anterior	–0.06	–6.80	–0.03	–4.00	0.03	4.10	0.07	8.21
Anteromedial	–0.01	–0.75	–0.01	–0.63	0.01	1.11	0.02	2.66
Posteromedial	0.04	4.94	0.02	2.95	–0.02	–2.94	–0.05	–5.78
Posterior	0.07	9.31	0.04	5.86	–0.04	–6.33	–0.09	–13.03
AVG	0.01	1.67	0.01	1.05	–0.01	–1.01	–0.01	–1.98
**M. gluteus minimus**								
Anterior	–0.08	–10.86	–0.05	–6.43	0.03	3.68	0.09	12.06
Medial	0.01	1.46	0.01	0.71	–0.04	–4.54	0.00	–0.17
Posterior	0.05	9.14	0.03	5.98	–0.10	–19.52	–0.07	–13.79
Avg	–0.01	–0.09	0.00	0.09	–0.04	–6.79	0.00	–0.63
*Lever arm*	(mm)	(%)	(mm)	(%)	(mm)	(%)	(mm)	(%)
**M. gluteus medius**	–6.42	–10.11	–4.12	–6.49	5.81	9.15	13.14	20.69
**M. gluteus minimus**	0.09	0.15	–0.87	–1.41	2.68	4.33	7.08	11.44

Abbreviation: FRDA, force ratio in the direction of abduction.

Anterior portions of the GMed muscle decreased in length after derotational osteotomy and increased in length after rotational osteotomy (−5.6% to +5.8%). Posterior portions showed the opposite (+7.2% to −8.7%). Averaged over all four branches, the muscle length of the entire GMed muscle remained almost balanced (±1.2%) after the simulated change in FT. GMin showed identical behaviour.

With a range of 1.7% increase for 20° of rotation and 2% decrease for 20° of derotation, the average FRDA for all GMed parts showed little relative change. The FRDA of the GMin showed a range of 0.1% increase for 10° of rotation and 7% decrease for 10° of derotation.

**Figure 4:**
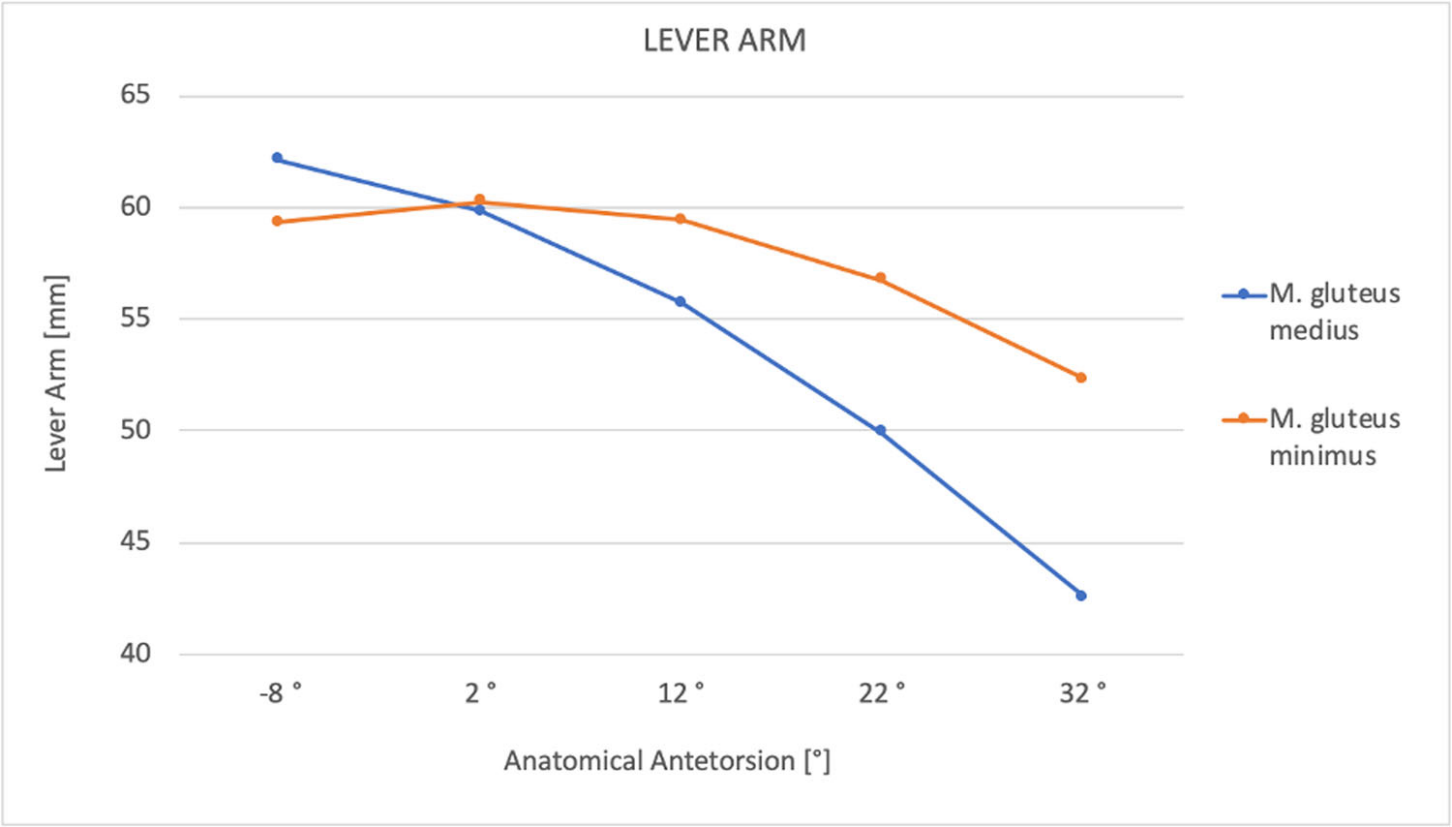
Relationship of the lever arm of the hip abductor muscles and anatomical femoral ante torsion. The maximum lever arm for gluteus medius and minimus muscles was 8° retrotorsion and 2° antetorsion, respectively.

The lever arm for GMed increased with derotation and decreased with rotation from +10.1% to −20.7%. The lever arm for GMin increased with derotation and decreased with rotation from +1.4% to −11.4%. The lever arm of each muscle in relation to the anatomical FT is shown in Figure 4.

## DISCUSSION

The most important finding of this study is that subtrochanteric osteotomy in treatment of femoral maltorsion affects the hip abductor moment solely through the altered lever arm. The influence of muscle length and FRDA on the hip abductor moment, on the other hand, is negligible. A 20° derotation for increased FT corresponds to a 20% increase in abduction force of the GMed (improved lever arm), whereas a 20° rotation for decreased FT reduces the abduction force by 10%.

The moment (abduction force) of a given muscle is defined by the muscle force, its lever arm, and the FRDA. According to Arnold et al. [1], the muscle force generation depends on the length of the muscle fibres relative to their optimum length. However, the muscle force‐length curve shows a plateau in muscle force development between 95% and 105% of the nominal muscle length, indicating no change in force generation. Given our results of muscle length differences of less than 2%, it can be assumed that the change in total muscle length after rotational or derotational osteotomy has no real effect on the hip abductor moment. The FRDA, which is linearly correlated with the abductor moment, also shows very small changes (<2%), so that the change in geometry of the abductor force insertion point after corrective osteotomies can be neglected. This leaves the lever arm as the only relevant factor influencing the hip abduction moment.

The results of our study show an increased hip abductor lever arm when FT is decreased, and vice versa. Recent literature [21, 22] has shown the same relationship between hip abductor lever arm and FT. In a 3D gait analysis of 30 healthy volunteers, De Pieri et al. [22] showed that the hip abductor lever arm ranged from 40 to 66 mm for 45° of antetorsion to 15° of retrotorsion. This is consistent with our results shown in Figure 3 (lever arm range of 42–63 mm).

The absolute muscle force is directly dependent on the lever arm, that is, a −10% reduction in the lever arm corresponds to a −10% abduction force from this muscle. Thus, a 20° derotation corresponds to a +20% and +10% change in abduction force for the GMed and GMin muscles, respectively. Patients with excessive FT will therefore experience an increase in the abductor lever arm as the GT is lateralised, which will increase muscular tension. In knees with a patellofemoral disease associated with excessive FT, the GT has been shown to be more anterior in relation to the femoral neck axis. Corrective osteotomy would result in a more anterior position of the GT as actually intended, but would mean an even less relevant influence on hip abductor strength [11]. Conversely, patients with reduced FT that are treated with a +20° rotational osteotomy due to femoroacetabular impingement can expect a 10% reduction in the GMed lever arm. The actual number may be slightly higher given the relationship of the GT to the anatomical torsion [2, 7] (relative posterior position of the GT and higher functional FT compared to anatomical FT).

The functional interpretation depends on how the leg is held in space by the patient. Basically, there are three possible factors that determine the position of the foot and therefore the GT: neuromotor memory, biomechanics or anatomy. First, the patient acquires a gait style over the years. This may be influenced by external factors such as the mother telling the patient to keep the foot straight when walking. As a result, the position of the foot is memorised and retained after the osteotomy. It could also mean that the patient would move with the femoral neck in the same orientation after the osteotomy, so that functionally there would be no translation of the GT, but an external or internal rotation of the foot after surgery. Hip abductor strength would remain the same. However, if the leg is positioned as biomechanically favourable as possible, then the leg will be held at 0° of FT so that the lever arm is the greatest—before or after surgery. The situation becomes even more complicated if there is anatomical compensation that allows the patient to walk without in/out‐toeing despite pathological FT [17]. In summary, it is very individual and therefore not possible to foresee how the leg will ultimately be held in space. For this reason, the figures presented here for GT repositioning, and therefore the change in lever arm, are calculated as maximum changes. Actual differences may be smaller because the leg is held differently in real life.

However, the big remaining question is whether changes of 10%–20% in abductor muscle strength fall below the threshold of clinical relevance. It is currently unknown what level of change would be required to be clinically relevant and therefore a plausible cause of muscle fatigue or lateral hip pain. However, in the light of recent findings in the literature on experimentally induced gluteal weakness, these effects can be considered small and clinically insignificant. In these studies, sequential nerve blocks of the tensor fascia latae, the GMed/GMin and gluteus maximus (GMax) were performed in healthy volunteers. Induced weakness of the GMed and Gmin reduced the abduction force by only 62%, as the GMax is highly relevant as an abductor of the hip [10]. The joint reaction force was reduced by 15%–45% during squatting in the study by Dimitriou et al. [4]. Nevertheless, this needs to be confirmed in future clinical trials. In addition, a longitudinal study would be needed to assess over time whether the change in abductor lever arm is associated with an increase/decrease in abductor‐related problems, as one would expect not only an immediate effect on functionality or gait with a change in muscle lever arm, but also a long‐term effect and degenerative changes in the muscle tendon. For the time being, and based solely on theoretical modelling, compensatory surgical procedures to address GT displacement can be argued to be unnecessary.

The main drawback of this study is that the results cannot be used to determine clinical relevance. This study provides important insights into the functionality of the hip abductors after subtrochanteric osteotomies and is one piece of a larger puzzle. Future studies will give adequate clinical relevance to the results of this study. Another limitation is that only static posture (bipedal upright stance) was analysed and not dynamic everyday movements such as walking, running or stair climbing. This is part of future projects. Furthermore, relative changes in hip abductor moment were calculated, and no conclusions can be drawn about the specific contribution or activation of the gluteal muscles to the absolute hip abduction moment. Finally, the use of an SSM represents a summation of the masses and may obscure individual anatomical features (e.g., coxa vara, coxa valga).

## CONCLUSIONS

Subtrochanteric osteotomy in the management of femoral maltorsion affects the hip abduction moment solely through the altered lever arm. A 20° derotation for increased FT corresponds to a 20% increase in abduction force of the GMed (improved lever arm), whereas a 20° rotation for decreased FT reduces the abduction force by 10%.

## AUTHOR CONTRIBUTIONS

**Christoph Zindel**: Conceptualization; data acquisition; formal analysis; investigation; methodology; project administration; software; validation; visualization; writing—original draft. **Patrick O. Zingg**: Formal analysis; writing—review and editing. **Michel Meisterhans**: Data acquisition; formal analysis; writing – review and editing. **Samuel Haupt**: Formal analysis; writing—review and editing. **Armando Hoch**: Conceptualization; supervision; writing—review and editing. **Andreas Flury**: Conceptualization; formal analysis; investigation; methodology; supervision; validation; writing—review and editing.

## CONFLICT OF INTEREST STATEMENT

The authors declare no conflicts of interest.

## ETHICS STATEMENT

Zurich Cantonal Ethics Commission, KEK‐ZH‐Nr 2017‐01616. All volunteers gave their informed consent.

## Data Availability

All data are available upon request by the author.
